# RAD-Deficient Human Cardiomyocytes Develop Hypertrophic Cardiomyopathy Phenotypes Due to Calcium Dysregulation

**DOI:** 10.3389/fcell.2020.585879

**Published:** 2020-10-22

**Authors:** Ya’nan Li, Yun Chang, Xiaolei Li, Xiaowei Li, Jian Gao, Yafei Zhou, Fujian Wu, Rui Bai, Tao Dong, Shuhong Ma, Siyao Zhang, Wen-Jing Lu, Xiaoqiu Tan, Yongming Wang, Feng Lan

**Affiliations:** ^1^Beijing Laboratory for Cardiovascular Precision Medicine, MOE Key Laboratory of Medical Engineering for Cardiovascular Diseases, MOE Key Laboratory of Remodeling-Related Cardiovascular Disease, Beijing Collaborative Innovation Center for Cardiovascular Disorders, Anzhen Hospital, Capital Medical University, Beijing, China; ^2^Beijing Institute of Heart, Lung and Blood Vessel Diseases, Beijing, China; ^3^Department of Cardiology, Heart Center, The First Affiliated Hospital of Xinxiang Medical University, Xinxiang, China; ^4^Experimental Medicine, Faculty of Medicine, Vancouver, BC, Canada; ^5^Key Laboratory of Medical Electrophysiology of the Ministry of Education, Institute of Cardiovascular Research, Southwest Medical University, Luzhou, China; ^6^The State Key Laboratory of Genetic Engineering and MOE Key Laboratory of Contemporary Anthropology, School of Life Sciences, Fudan University, Shanghai, China; ^7^State Key Laboratory of Cardiovascular Disease, National Center for Cardiovascular Diseases, Fuwai Hospital, Chinese Academy of Medical Sciences and Peking Union Medical College, Beijing, China

**Keywords:** *RRAD* knockout, RAD deficiency, HCM, L-type Cacpsdummy2+ channels, calcium handling, calcium channel blocker

## Abstract

Ras associated with diabetes (RAD) is a membrane protein that acts as a calcium channel regulator by interacting with cardiac L-type Ca^2 +^ channels (LTCC). RAD defects can disrupt intracellular calcium dynamics and lead to cardiac hypertrophy. However, due to the lack of reliable human disease models, the pathological mechanism of RAD deficiency leading to cardiac hypertrophy is not well understood. In this study, we created a *RRAD*^–/–^ H9 cell line using CRISPR/Cas9 technology. RAD disruption did not affect the ability and efficiency of cardiomyocytes differentiation. However, RAD deficient hESC-CMs recapitulate hypertrophic phenotype *in vitro*. Further studies have shown that elevated intracellular calcium level and abnormal calcium regulation are the core mechanisms by which RAD deficiency leads to cardiac hypertrophy. More importantly, management of calcium dysregulation has been found to be an effective way to prevent the development of cardiac hypertrophy *in vitro*.

## Introduction

Cardiac hypertrophy is a significant adaptive change in response to various stimuli from inside and outside the body. Physiological hypertrophy can preserve cardiac function, while pathological hypertrophy is often accompanied by some adverse events, such as arrhythmia, heart failure, sudden cardiac death (SCD), thus becomes an independent risk factor for cardiac mortality (Shimizu and Minamino, 2016; Nakamura and Sadoshima, 2018). It is said that some regulatory genes related to calcium handling have been found to be involved in the pathogenesis of cardiac hypertrophy (Chang et al., 2007).

Ras associated with diabetes (RAD), a membrane protein consists of 308 amino acids, is encoded by human *RRAD* gene and is highly expressed in cardiomyocytes (Reynet and Kahn, 1993; Maguire et al., 1994). It acts as a calcium channel regulator by interacting with cardiac L-type Ca^2 +^ channels (LTCC), which play a fundamental role in normal heart (Finlin et al., 2003). Previous evidence has shown that elevated intracellular Ca^2 +^ and abnormal calcium regulation are the central mechanism for inducing cardiac hypertrophy (Lan et al., 2013; Monteiro da Rocha et al., 2016; Dewenter et al., 2017; Li et al., 2019). Studies on mice indicated that deficiency of RAD function in cardiomyocytes, which can lead to an increased L-type Ca^2 +^ current (*I*_Ca–L_) via upregulation of LTCC expression in the plasma membrane (Yada et al., 2007), significantly increased stress-induced cardiac hypertrophy and remodeling *in vitro* (Chang et al., 2007) and cardiac fibrosis *in vivo* (Zhang et al., 2011). In addition, RAD is significantly decreased in human failing hearts (Chang et al., 2007). Recently, a new study identified a rare missense *RRAD* mutation (p.R211H) in Brugada syndrome patient, which can disturb Na^+^ current (*I*_Na_) and *I*_Ca–L_ thus leads to structural and electrical defects in cardiomyocytes (Belbachir et al., 2019). However, due to the lack of a human heart disease model, the role of RAD functional defects in the human heart is unclear.

Although efforts over past few decades have revealed the molecular function and pathogenic mechanism of RAD to a certain extent, these results are basically derived from the mouse model. However, due to species differences, cardiac electrophysiological characteristics are significant different between humans and mice. For example, mouse have a higher resting heart rate (500–700 bpm), a more negative action potential plateau, and a shorter action potential duration (APD) compared to human (Watanabe et al., 2011). Thus, mouse models could not effectively mimic the pathological process of human heart diseases. It is of great significance to establish a reliable human heart disease model to study RAD function. Fortunately, the rapid development of human pluripotent stem cells (PSCs) technology in recent years has provided a powerful tool for studying human cardiovascular diseases (Monteiro da Rocha et al., 2016; Chelko et al., 2019; Park et al., 2019).

Thus, we created a human embryonic stem cell line (hESCs) with RAD deficiency (*RRAD*^–/–^) using CRISPR/Cas9 technology to explore the function of *RRAD* gene. After differentiated both wild type (WT) and *RRAD*^–/–^ hESCs (KO) into cardiomyocytes *in vitro*, we were surprised to find that cardiomyocytes derived from *RRAD*^–/–^ hESCs exhibited a distinct hypertrophic phenotype compared to wild type. Further studies confirmed that abnormal regulation of intracellular calcium level may be a major mechanism of cardiac hypertrophy. Based on the results, early interventions for abnormal calcium handling can prevent this pathological process.

## Materials and Methods

### Human Embryonic Stem Cell Culture and Cardiac Differentiation

This study was approved by the Ethics Committee of Anzhen Hospital, Capital Medical University (#134/18). Human embryonic stem cell H9 (hESC-H9) were cultured on Matrigel-coated (Corning, United States) feeder-free plates with E8 medium (Cellapy, China). Cells were changed with fresh medium every day and passaged routinely using 0.5 mM EDTA without MgCl_2_ or CaCl_2_ (HyClone, United States) when confluence reaches 80%. Cells were maintained at 37°C, 5% CO_2_ incubator.

Human embryonic stem cell H9 were differentiated into cardiomyocytes using a chemically defined small molecule-based protocol as previously reported (Burridge et al., 2014). Purification of cardiomyocytes using a metabolic-selection method as previous described (Tohyama et al., 2013).

### Genome Editing

Single guide RNA (sgRNA) (TGCAGGTCGCGCTCGTCCAC) targeting *RRAD* gene were designed for next gene knock-out. The sgRNA was then ligated into the epiCRISPR plasmid as previously described (Xie et al., 2017). After disassociated into single cells, about 1 × 10^6^ H9 cells were electroporated with 2–5 μg epiCRISPR plasmid containing the sgRNA using 4D nucleofector system (Lonza, Germany). Cells were then cultured with E8 medium supplemented with 10 μM Y-27632 (Rho kinase inhibitor) at the first 24 h and then selected with puromycin for 3–5 days. The positive clones were picked into 24-well plate for sequencing identification.

The protocol of both H9 and H9-*RRAD*^–/–^ GCaMP reporter cell lines were generated as previously reported (Li et al., 2019).

### Immunostaining and Imaging Analyses

Cells were fixed with 4% PFA for 30 min, permeabilized with 0.5% Triton X-100 (Sigma, United States) for 15 min, and blocked with 3% BSA (Sigma) for 30 min. Cells were then incubated with primary antibodies overnight. After washed with PBS 3 times, cells were then incubated with secondary antibodies for 45 min at 37°C and counterstained with DAPI (Invitrogen, United States) for 10 min. Fluorescence images were performed under Leica DMI 4000B. Both primary and secondary antibodies were listed at an appropriate dilution in Supplementary Table S2.

### Flow Cytometry

Both WT and KO cardiomyocytes were dissociated into single cells using CardioEasy CM dissociation buffer (Cellapy), and fixed with chilled fixation buffer (BD Biosciences) for 10–15 min at 25°C. Fixed cardiomyocytes were incubated with anti-cardiac troponin T antibody (cTnT) and then Alexa Fluor 488 secondary antibodies for 30 min, respectively. Cardiomyocytes were then washed with PBS three times and analyzed using FACS analysis equipment (EPICS XL, Beckman). Data were analyzed with FlowJo X software.

For multinuclear detection experiments, the cells were fixed with chilled 70% ethanol at −20°C for 24 h, washed with PBS one time, stained with 50 μg/mL propidium iodide (Becton, Dickinson and Company, Franklin lake, NJ, United States) at room temperature for 15–20 min and analyzed using EPICS XL (Beckman Coulter). Data were analyzed with Modfit LT Software.

### Cellular Ca^2 +^ Imaging

Cardiomyocytes derived from H9-GCaMP and *RRAD*^–/–^-GCaMP lines were seeded onto Matrigel-coated confocal dishes or eight-well chambers. Intracellular Ca^2 +^ flux was imaged at 40× using a confocal microscope (Leica, TCS5 SP5, Germany). Spontaneous intracellular Ca^2 +^ transients were recorded at 37°C, 5% CO_2_ using standard line-scan mode. A total of 8192 line-scans were acquired for 8.192 s recording durations. For caffeine-induced Ca^2 +^ release, 20 μM caffeine was used to release SR Ca^2 +^. Results were analyzed using Image J and Igor software.

### RNA Extraction and Quantitative Real-Time Polymerase Chain Reaction

Total RNA was isolated from (0.5−1) × 10^6^ cells with TRIzol Reagent (Life Technologies, United States) and then treated with DNase I (Life Technologies) for 30 min at 37°C to remove DNA contamination. cDNA was prepared using the PrimeScript^TM^ reverse transcription system (Takara, Japan) as manufacturer’s instructions. The gene expression levels were examined by quantitative real-time polymerase chain reaction (qRT-PCR) using the iCycler iQ5 (Bio-Rad) with 2 × SYBR Master Mix (Takara) and the relative quantification was analyzed according to the ΔCT method. All primer sequences used were listed in Supplementary Table S1.

### RNASeq Processing and Biological Information Analysis

Construction and sequencing of transcriptome libraries were executed by Annoroad Gene Technology Corporation (Beijing, China). Quality-qualified libraries were selected for sequencing using the Illumina platform. The sequencing strategy was PE150. The Raw Reads sequence from the Illumina platform was sequenced to obtain high-quality sequences (Clean Reads) by de-sequencing low-quality sequences and de-junction contamination, etc. All subsequent analyses were based on Clean Reads. Gene differential expression analysis were performed using DESeq2. The differential gene screening was mainly based on the difference fold (Fold change value) and the *q* value (padj value, *P* value after correction). Differential genes with |log2 Fold change| ≥ 1 and *q* < 0.05 were usually selected as differentially expressed genes (DEGs). Heatmaps of hierarchical clustering analysis of DEGs was performed using R-package. Gene ontology (GO) enrichment analysis was performed on these DEGs, and a false discovery rate (FDR) of less than 0.05 was considered to be significantly enriched. For KEGG functional analysis, enrichment analysis was performed on each pathway using hypergeometric tests to identify pathways for significant enrichment in DEGs.

### Electrophysiological Recording

L-type calcium current (*I*_Ca–L_) in hESCs-CM was recorded by using EPC-10 amplifier system (HEKA Elektronik, Lambrecht, Germany) under whole-cell patch-clamp configuration. Firstly, hESCs-CMs were plated onto 13-mm glass coverslips and current recording was performed 48–72 h later. For recording of the *I*_Ca–L_ in hESCs-CMs, the bath solution contained (in mM): 140 TEA, 5 BaCl_2_ and 10 HEPES adjusted to pH 7.3 with CsOH. The pipette solution contained (in mM): 145 CsCl, 145 Cs-MeSO_3_, 1 MgCl_2_, 4 Mg-ATP, 0.5 EGTA and 10 HEPES, adjusted to pH 7.3 with CsOH. Series resistance (Rs) was compensated by about 70% and less than 10 MΩ to minimize voltage errors. *I*_Ca–L_ was measured at the holding potential of −80 mV followed by 300 ms depolarization test pulse steps from −50 to +60 mV in 10 mV increments with 1 s test interval. 200 nM nifedipine, a specific blocker of L-type voltage-gated Ca^2 +^ channels, was added to the external solution to identify the current. All experiments were performed at room temperature, 22 ± 1°C.

### Western Blot Analysis

Cells to be tested were washed with pre-cooled PBS 3 times and then lysed with Mammalian Protein Extraction Reagent (Thermo, #78501, United States) containing 5 mM EDTA (Thermo, #1861275), protease inhibitor cocktail (Thermo, #1861278) and phosphatase inhibitor cocktail (Thermo, #1862495). Cell lysates collected were then vibrated three times every 10 min and centrifuged at 12,000–15,000 rpm for 15 min. For the extraction of membrane proteins, we used the Membrane Protein Extraction Kit (Thermo, #89842, United States) following the manufacturer’s instructions. The concentration of supernatant protein was determined by Pierce^TM^ BCA Protein Assay Kit (Thermo, #23227). Next, 20–40 ug denatured protein samples were detected by electrophoresis on 6–12% [depending on the molecular weight of the protein to be tested. For proteins with a molecular weight greater than 250 KD, we used 6% sodium dodecyl sulfate-polyacrylamide gels electrophoresis (SDS-PAGE), added 0.5% SDS to the electrophoretic buffer, and reduced the methanol concentration to 10%]. SDS-PAGE and transferred to PVDF membrane or nitrocellulose membrane at 300 mA for 90–180 min (depending on the molecular weight of the protein to be tested) using gel transfer device (Bio-Rad). After blocked with 5% skimmed milk powder for an hour at room temperature, membranes were incubated with primary antibodies at 4°C overnight and then detected with corresponding secondary antibody. Both primary and secondary antibodies were listed in the Supplementary Table S2 at an appropriate concentration.

### Statistical Analysis

All experiments were repeated at least three times and data were shown as mean ± standard errors of the means (S.E.M.). Statistical comparisons were determined using two-sided Student’s *t*-test between two groups or one-way ANOVA tests followed by Tukey’s Multiple Comparison Test among multiple groups. Statistically significant was determined as a value of *P* < 0.05.

## Results

### Establishment of *RRAD*^–/–^ hESC Line

**FIGURE 1:**
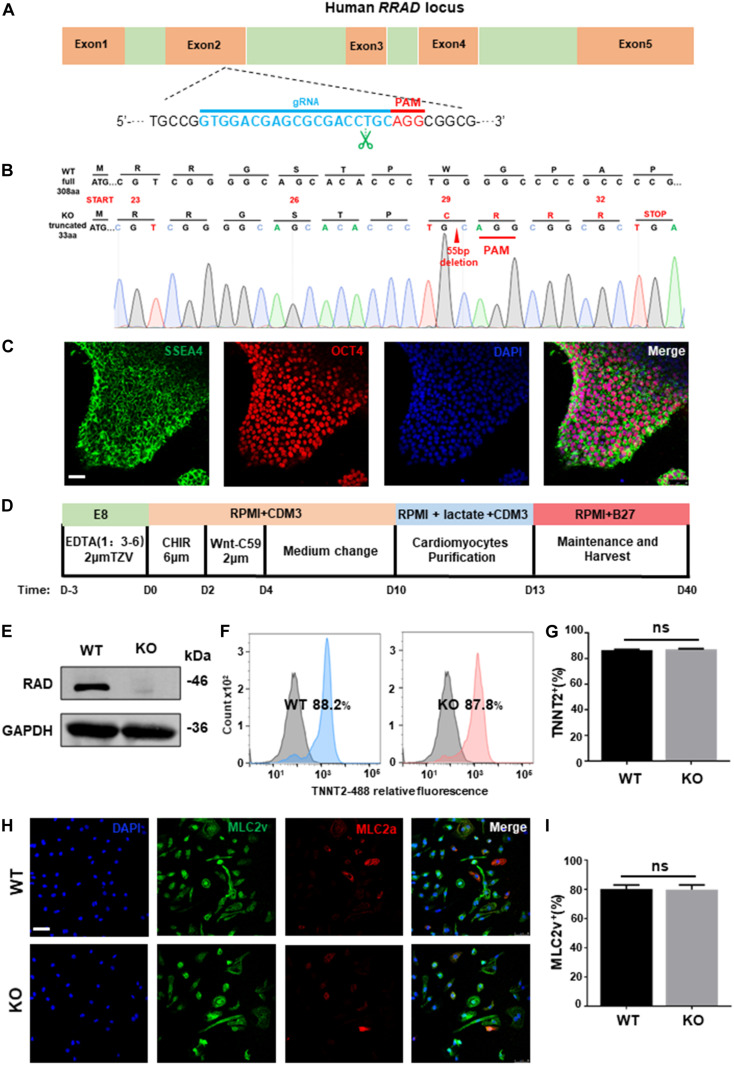
*RRAD* knockout did not affect the pluripotency nature of hESC and the ability of cardiac differentiation. **(A)** Schematic of human *RRAD* locus and editing site of designed gRNA-epiCRISPR/Cas9. **(B)** Sequencing data shows deletion of 55 bases in *RRAD* knockout hESC lines. **(C)** Immunostaining for the pluripotency markers SSEA4 and OCT4 of *RRAD* knockout hESC lines. Scalebar, 50 μm. **(D)** Schematic of cardiac differentiation using standard small molecule-based protocols. **(E)** Western blot shows RAD expression in WT and KO CMs at days 15. **(F,G)** Flow Cytometry for TNNT2 staining in representative WT and KO CMs at days 10 without purification. **(H,I)** Immunostaining for protein expression of MLC2v and MLC2a in WT and KO CMs at days 30. Scalebar, 50 μm. Data are expressed as means ± S.E.M. of three independent experiments. ns, not significant.

To establish a genetical platform for exploring the function of *RRAD* gene, a sgRNA targeting exon 2 of *RRAD* were designed (Figure 1A). After electroporated with plasmid (containing sgRNA and epiCRISPR/Cas9) and selected with puromycin, we screened twelve clones for genotyping. Among these, one was no editing, six were heterozygous (with an unedited wild type allele) and five were homozygous (biallelic mutations). However, of the five homozygous, the sequencing results of two clones showed no frameshift mutations (one was edited with −21 bp and the other with +3 bp), so it was not used in the next experiment (data not shown). The sequencing results of the other three homozygous were: #7: −55 bp; #9: −14 bp; #12: +1 bp, −8 bp (Figure 1B and Supplementary Figure S1A). We selected #7, with 55 base pairs deletion resulting in frame-shifted coding sequence, for subsequent research (Figure 1B). Besides, in order to determine whether the *RRAD* gene knockout played the same role in different stem cell lines, we also established a *RRAD*^–/–^ hESCs-NKX2-5-GFP cell line using the same method. We selected five clones for genotyping. Among these, two were no editing, two were heterozygous (with an unedited wild type allele) and one were homozygous (biallelic mutations). This homozygous was edited by inserting 7 base pairs near the PAM region, causing a frameshift mutation (Supplementary Figure S1B). In addition, we analyzed the top ten off-target sites of sgRNA with online analysis software^1^, and no off-target mutations were found identified by DNA sequencing (data not shown) (Pankowicz et al., 2016). *RRAD*^–/–^ line exhibited normal morphology (Supplementary Figure S1C), expressed pluripotency markers OCT4 and SSEA4 (Figure 1C), and had no chromosomal abnormalities (Supplementary Figure S1D). Teratoma formation assay generated cellular derivatives of three germ layers *in vivo* indicated that *RRAD* knockout did not affect the pluripotent nature of hESCs (Supplementary Figure S1E).

### Differentiation of *RRAD*^–/–^ hESCs Into Cardiomyocytes

Both established *RRAD*^–/–^ line and WT hESCs were then differentiated into cardiomyocyte lineages (hESC-CMs) using standard small molecule-based protocols (Figure 1D). From 7 days after initiation of differentiation, spontaneous beating could be detected in WT (60 ± 5 beats per min) and KO (57 ± 7 beats per min) cardiomyocytes and no significantly difference in beating rates between the two groups (Supplementary Figures S2A,B). At day 15, we identified by western blot that cardiomyocytes derived from *RRAD*^–/–^ line exhibited complete loss of RAD protein (Figure 1E). Flow cytometry for cardiac Troponin T indicated that cardiomyocytes both derived from WT and KO were reached more than 85% (Figures 1F,G). Immunostaining for MLC2v and MLC2a demonstrated that about 80% cardiomyocytes of both WT and KO were positive for ventricular-specific marker MLC2v (Figures 1H,I). Taken together, these findings suggested that *RRAD* knockout did not affect the ability and efficiency of myocardial differentiation.

### *RRAD*^–/–^ Cardiomyocytes Recapitulate Hypertrophic Phenotype *in vitro*

**FIGURE 2:**
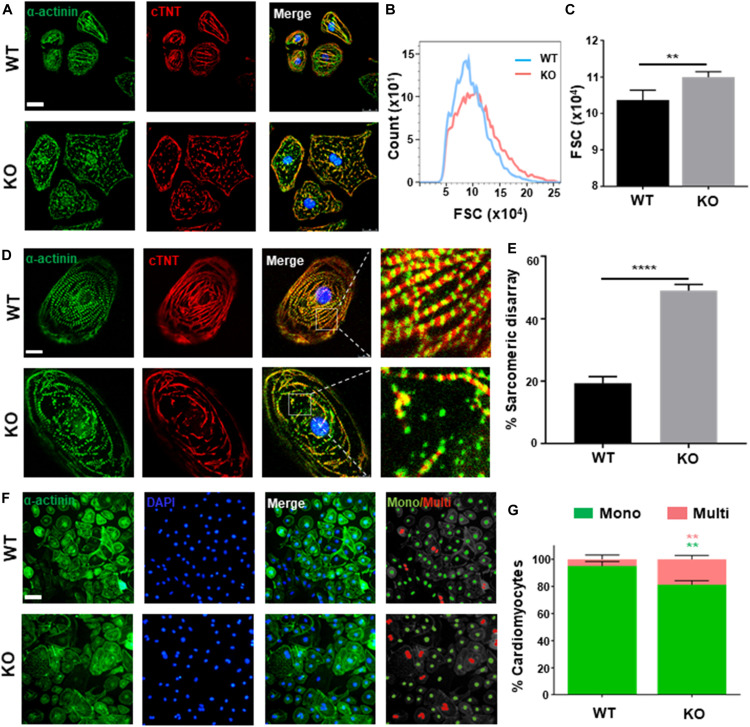
Phenotyping cardiac hypertrophy in RAD deficient CMs. **(A)** Images of α-actinin (green) and cTnT (red) immunostaining show an increased size in KO CMs at days 40. Scale bar, 50 μm. **(B,C)** Calibration of forward scatter (FSC;10000 cells/sample, *n* = 3) shows an increased size in KO CMs at days 40. **(D)** Images of α-actinin (green) and cTnT (red) immunostaining show disorganized arrangement of myofibrils in KO CMS. Scale bar, 50 μm. **(E)** Quantification of disorganized myofibrils in WT (*n* = 206) and KO CMs (*n* = 198). **(F)** Images of α-actinin/DAPI immunostaining. Scale bar, 50 μm. **(G)** Quantification of mono- and multi-nucleation in WT (*n* = 236) and KO (*n* = 262) CMs. Data are expressed as means ± S.E.M. of three independent experiments. **P < 0.01; ****P < 0.0001.

Clinically, dysfunction in cardiac hypertrophy is mainly owing to thickening ventricular wall and cardiomyocytes hypertrophy (Harvey and Leinwand, 2011). As present study shows, both hESC-H9 and hESCs-NKX2-5-GFP-derived *RRAD*^–/–^ cardiomyocytes were noticeably larger than WT at day 40 post-induction (Figure 2A and Supplementary Figure S2C). Further verification by flow cytometry, measured with forward scatter of calibration spheres, also validated this conclusion (Figures 2B,C). In addition to increased size of cardiomyocytes, multi-nucleation (Arad et al., 2002) and disorganized arrangement of myofibrils (Kraft et al., 2013; Tanaka et al., 2014) are also common features of cardiac hypertrophy. Further immunofluorescence staining for cardiac Troponin T and α-actinin exhibited significantly higher frequencies of myofibrillar disarray (Figures 2D,E and Supplementary Figure S2C) and multinucleation (Figures 2F,G) in *RRAD*^–/–^ cardiomyocytes compared to WT. Also, flow cytometry shows an increased percentage of tetraploid and hexaploid in *RRAD*^–/–^ cardiomyocytes (Supplementary Figure S2D). Taken together, these findings suggested that cardiomyocytes derived from *RRAD*^–/–^ line could recapitulate hypertrophic phenotype *in vitro*.

### Differential Transcriptome of *RRAD*^–/–^ Cardiomyocytes

**FIGURE 3:**
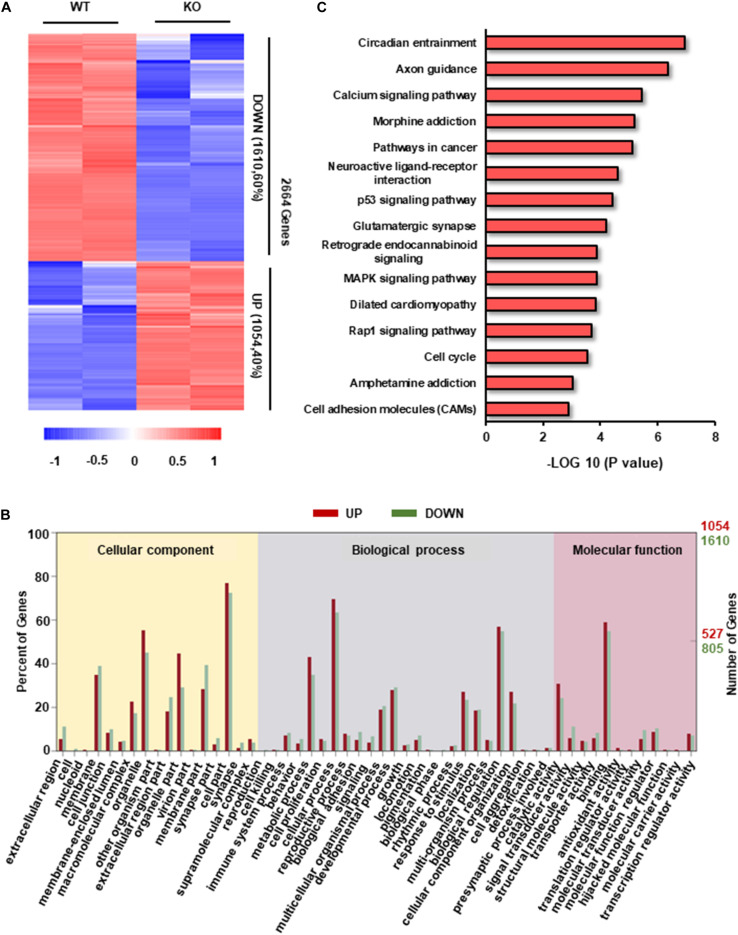
Transcriptome analysis in RAD deficient and WT CMs. **(A)** Heatmap of transcripts from WT and KO CMs (*n* = 2, respectively) show consistency within groups. Red: upregulated transcripts. Blue: downregulated transcripts. **(B)** Gene ontology (GO) term categories and distribution of differentially expressed genes. GO terms are divided into three categories: cellular component, molecular function and biological process. **(C)** Significantly enriched Kyoto Encyclopedia Genes and Genomes (KEGG) pathways in KO CMs.

To explore the potential molecular mechanisms of hypertrophic phenotype caused by RAD deficiency, we examined the transcriptome characteristics of *RRAD*^–/–^ cardiomyocytes in comparison to WT cardiomyocytes. We found a total of 2664 transcripts differentially expressed between *RRAD*^–/–^ and WT cardiomyocytes, from which, 1054 transcripts were upregulated, and 1610 transcripts were downregulated (Figure 3A). After GO enrichment analysis of differentially expressed transcripts, we identified 17, 25, and 12 categories in cellular component, biological process, molecular function, respectively (Figure 3B and Supplementary Figures S3A–C). Among them, ion binding and biological regulation were the most differentiated categories within molecular function and biological process, respectively, indicated that RAD plays a vital role in biological regulation and closely related to the function of ions, which is consistent with previous researches (Figure 3B and Supplementary Figures S3A–C; Yada et al., 2007; Belbachir et al., 2019). To explore the biological impacts of these differentially expressed transcripts, we next performed KEGG (Kyoto Encyclopedia of Genes and Genomes) functional analysis. Results as shown in Figure 3C, although “circadian entrainment” and “axon guidance” rank first and second in GO analysis of 15 significantly different pathways, they were involved in circadian rhythm control of organism (Golombek and Rosenstein, 2010) and neurological disorders (Van Battum et al., 2015), respectively. We focused on the third “calcium signal pathway” for it is closely related to the physiological function of *RRAD* gene and it has been confirmed as the central mechanism of cardiac hypertrophy in previous studies (Lan et al., 2013; Dewenter et al., 2017; Li et al., 2019). Taken together, these findings suggested that RAD is involved in the regulation of biological processes and is closely related to the regulation of calcium ion function.

### *RRAD*^–/–^ Cardiomyocytes Display Abnormal Regulation of Intracellular Calcium

**FIGURE 4:**
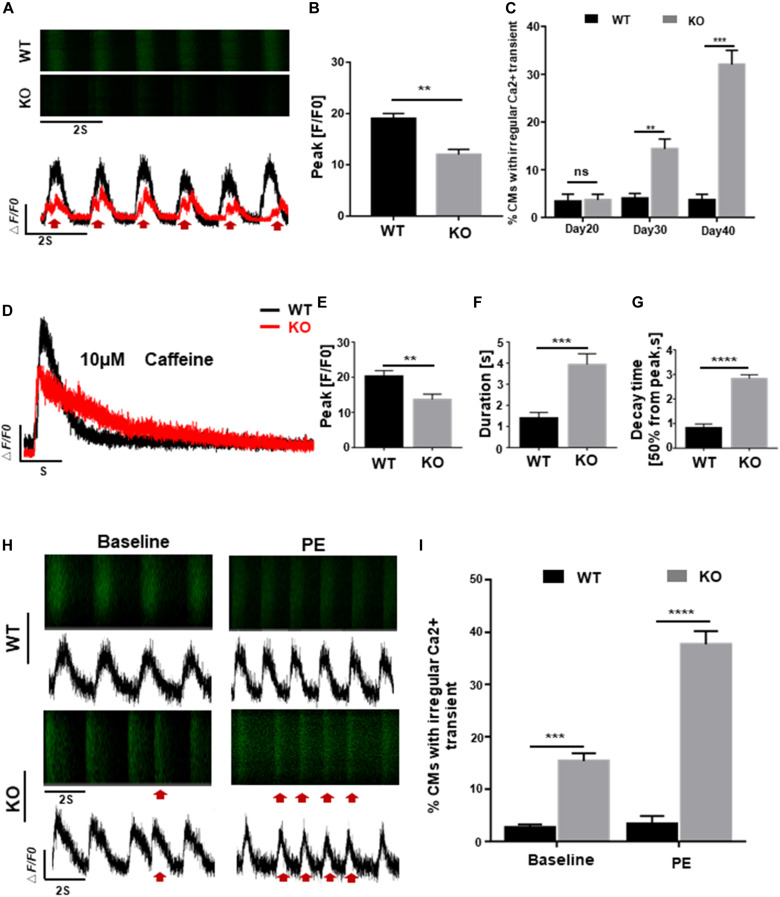
RAD deficient CMs display abnormal regulation of intracellular calcium. **(A)** Representative line-scan images in WT and KO CMs. Red arrows indicate arrhythmia-like voltage waveforms observed in KO CMs but not in WT. **(B)** Peak amplitudes in WT and KO CMs (*n* = 30 cells per group). **(C)** Quantification of percentages for WT and KO CMs exhibit abnormal calcium regulation at days 20, 30, and 40 (*n* = 30 cells per group). **(D)** Representative Ca^2 +^ transient from WT and KO CMs induced by caffeine exposure (*n* = 5 cells per group). **(E–G)** Peak amplitudes, duration and decay time after caffeine-induced Ca^2 +^ transient in WT and KO CMs. **(H)** Representative line-scan images in WT and KO CMs after adrenergic stimuli by PE. Red arrows indicate arrhythmia-like voltage waveforms. **(I)** Quantification of WT and KO CMs exhibit arrhythmia-like voltage waveforms in response to PE. Data are expressed as means ± S.E.M. of three independent experiments. ns, not significant; **P < 0.01; ***P < 0.001; ****P < 0.0001.

Homeostasis of calcium activity is crucial for cardiac excitation-contraction (EC) coupling and electrophysiological properties, early disruption of which, however, can lead to various dysfunctions such as cardiac hypertrophy and arrhythmia (Bers, 2008; Monteiro da Rocha et al., 2016). To explore the possible effects of RAD deficiency on calcium regulation, we generated GCaMP reporter cells as previous reported (Li et al., 2019) and assessed calcium transient of cardiomyocytes in both WT and KO lines at days 20, 30, and 40 post-induction. Results as shown in Figures 4A,B, cardiomyocytes derived from KO line showed smaller amplitude and significantly altered calcium transient which may be associated with arrhythmia-like voltage waveforms compared to control cells. Furthermore, abnormal calcium transients were observed at day 30 in KO CMs prior to the onset of hypertrophic phenotype, indicating that abnormal regulation of intracellular calcium level may be a critical factor in the pathogenesis of cardiac hypertrophy at cellular level (Figure 4C). Measurement of sarcoplasmic reticulum (SR) calcium stores induced by caffeine showed lower level of calcium release and prolonged decay time in KO CMs further prove that calcium dysregulation is occurring in the cell, which is consistent with the results above (Figures 4D–G and Supplementary Figure S3D).

Besides, fatal cardiac events in some patients with cardiac hypertrophy are often triggered by adrenergic stimuli, we administrated 10 μmol/L phenylephrine (PE) to both WT and KO CMs to mimic the stimulation. After 5 days of treatment, severely exacerbate presentation of multiple events were more frequently observed in KO CMs compared to WT which only showed an increased beating rate without rhythm disturbance (Figures 4H,I). These findings suggested that cardiomyocytes derived from *RRAD*^–/–^ line were more susceptible to phenylephrine-induced arrhythmias, one of the clinical manifestations of patients with cardiac hypertrophy.

### *RRAD*^–/–^ Cardiomyocytes Exhibit Activation of Hypertrophy-Associated Genes

**FIGURE 5:**
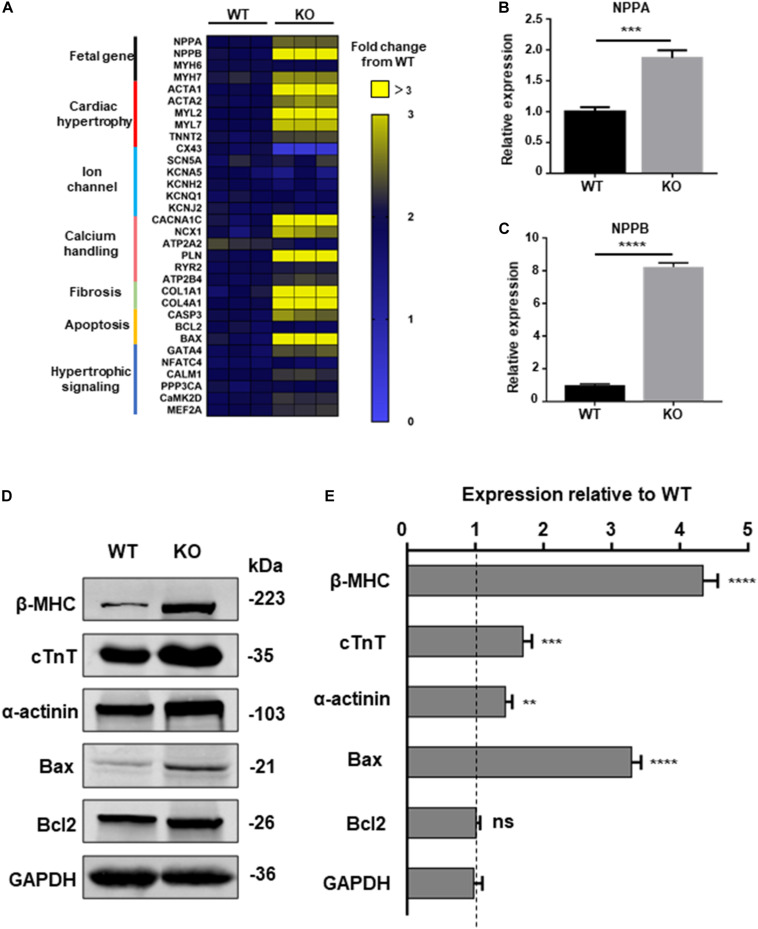
Increased hypertrophic signaling in RAD deficient CMs. **(A)** Heatmap shows expression changes of genes related to cardiac hypertrophic signaling pathways in WT and KO CMs at days 40. **(B,C)** Specific markers for cardiac hypertrophy *NPPA*, *NPPB* are significant upregulated in KO CMs. **(D)** Western blot shows expression of hypertrophic related proteins in WT and KO CMs, respectively. **(E)** Quantification of protein expression normalized by GAPDH in WT and KO CMs. Data are expressed as means ± S.E.M. of three independent experiments. ns, not significant; **P < 0.01; ***P < 0.001; ****P < 0.0001.

To further explore the relationship between dysfunction of RAD and pathological process of cardiac hypertrophy, a panel of genes involved in hypertrophy include cardiac structure and function were measured by qPCR at day 20, 30, and 40 post-induction (Narsinh et al., 2011). Beginning at day 40, cardiomyocytes derived from KO line presented remarkably increased mRNA expression of fetal program, hypertrophy, hypertrophic signaling, fibrosis, calcium handling and apoptosis (*CASP3*, *BAX*) which has been identified to be closely related to cardiac hypertrophy (Figure 5A and Supplementary Figure S4A; Mosqueira et al., 2018). ANF, BNP, markers of cardiac hypertrophy, respectively encoded by *NPPA*, *NPPB*, were significant upregulated in KO CMs (Figures 5B,C; Lowes et al., 1997; Gardner, 2003). Meanwhile, results of western blot exhibited increased protein expression of cardiac structure and apoptosis-associated proteins which matches the trend of the qPCR result (Figures 5D,E). Also, the results of trypan blue staining suggested an increased proportion of dead cardiomyocytes in the KO group, which may be a result of an increase in pro-apoptotic factors (Supplementary Figures S5A,B).

### *RRAD* Knock-Out Increased LTCC Expression and *I*_Ca–L_ in the Cardiomyocytes

**FIGURE 6:**
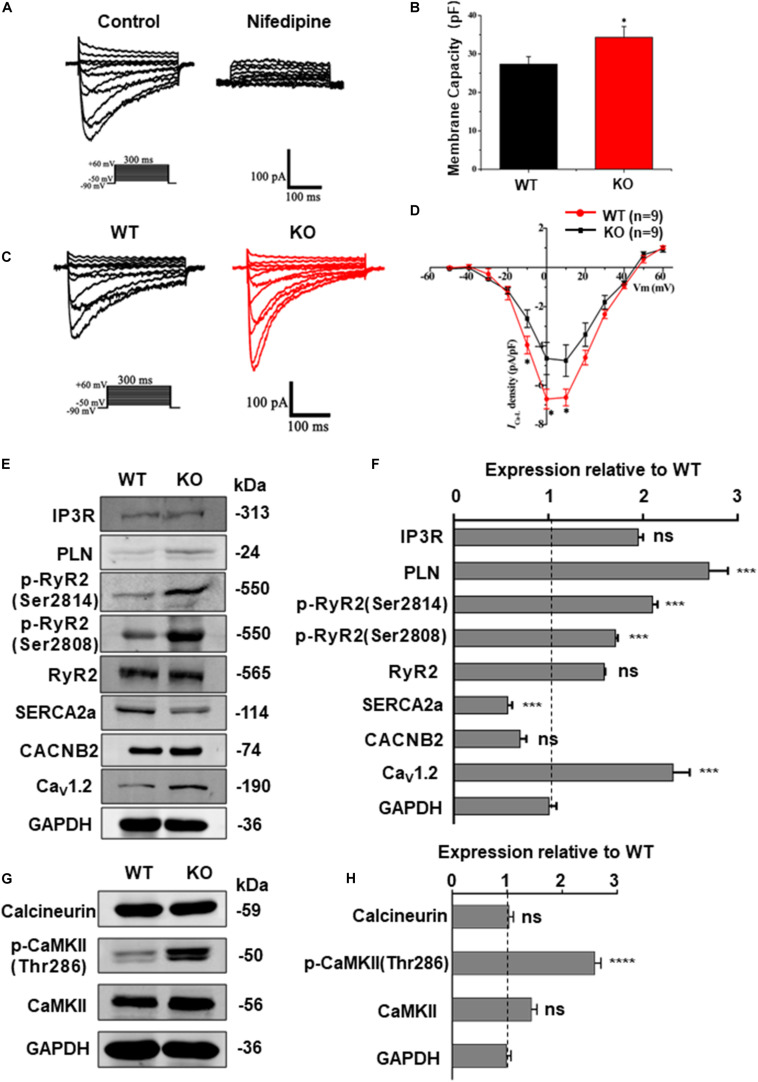
RAD deficient CMs show increased *I*_Ca–L_ and CaMKII activity. **(A)** Recording and identification of representative *I*_Ca–L_ from a series of step pulses between −50 mV to +60 mV from a holding potential of −80 mV. 200 nM nifedipine, a specific blocker of L-type voltage-gated Ca^2 +^ channels, blocked the current. **(B)** Histogram analysis showing that the cell membance capacity (pF) was larger in KO CMs than that in WT CMs. **(C)** Typical *I*_Ca–L_ recording from WT CMs (black traces) and KO CMs (red traces). **(D)** I-V curves *I*_Ca–L_ showing that *I*_Ca–L_ *density* was significantly increased in KO CMs (*n* = 9, respectively). **(E)** Western blot shows expression of protein related to calcium regulation in WT and KO CMs, respectively. **(F)** Quantification of protein expression normalized by GAPDH in WT and KO CMs. **(G)** Western blot shows expression of calcineurin A, CaMKIIδ and phosphorylated CaMKII (Thr286) in WT and KO CMs, respectively. **(H)** Quantification of protein expression normalized by GAPDH in WT and KO CMs. Data are expressed as means ± S.E.M. of three independent experiments. ns, not significant; *P < 0.05; ***P < 0.001; ****P < 0.0001.

The homeostasis of intracellular Ca^2 +^ concentration is maintained by a common regulation of the membrane and cytoplasmic transport systems. Among these, LTCC is the most important type of calcium channel located in the membrane of cardiomyocytes. Previous studies on mice have demonstrated that deficiency of RAD can lead to an increase of *I*_Ca–L_ via upregulation of LTCC expression in the plasma membrane (Yada et al., 2007). To determine the effect of *RRAD* gene knockout on *I*_Ca–L_ in cardiomyocytes derived from hESCs, we subsequently performed whole-cell patch-clamp experiments. Results as shown in Figures 6A–D, cardiomyocytes derived from KO line showed significantly increased membrane capacitance (pF) (Figures 6A,B). I-V curves *I*_Ca–L_ from the cardiomyocytes in each group show that *I*_Ca–L_ density (pA/pF) was significantly increased in KO CMs (Figures 6C,D).

Besides, western blot was performed to quantify the expression of Ca_v_1.2 (α1c subunit of LTCC, encoded by *CACNA1C* gene) and a series of proteins closely related to intracellular calcium regulation, including RyR2, phosphorylated RyR2 (RyR2-Ser2808, RyR2-Ser2814), SERCA2a, PLN, IP3R and CACNB2 (β2 subunits of LTCC). Results as shown in Figures 6E,F, there was no difference in the expression of CACNB2 between two groups, but the expression of Ca_v_1.2 was significantly increased in the KO CMs, which can be regarded as a potential evidence of increased intracellular calcium levels and could be correlated to various pathological process. In order to further detect the expression of Ca_v_1.2 protein on the cardiomyocyte membrane, we extracted the total membrane protein of the WT and KO CMs. Result as shown in Supplementary Figures S4B,C, the expression of Ca_v_1.2 was also significantly increased in the KO CMs. In addition, there was no difference in the expression of RyR2 between the two groups, while the expression of p-RyR2 (RyR2-Ser2808, RyR2-Ser2814) increased in the KO CMs, the expression of SERCA2a decreased, and the expression of PLN, which negatively regulates SERCA2a, also increased (Figures 6E,F). These results indicate that although the calcium released by SR increases, it cannot be effectively recovered due to impaired SERCA2a function, which explains the decrease of SR calcium to a certain extent and also becomes another important factor for the increase of intracellular calcium load. Furthermore, results of western blot at protein level were also consistent with the transcripts level of *CACNA1C*, *CACNB2*, *RYR2*, *ATP2A2*, *PLN*, and *ITPR1* (Supplementary Figure S6A).

### *RRAD* Knock-Out Increased CaMKII Activity in the Cardiomyocytes

Calcium is a universal intracellular second messenger and can interact with a variety of intracellular molecules (Bers, 2008). Among these, CaMKII (Ca^2 +^/calmodulin-dependent kinase II) and calcineurin (Ca^2 +^/calmodulin-dependent serine/threonine phosphatase calcineurin) have been widely studied on their role in cardiac hypertrophy (Dewenter et al., 2017). Moreover, our data have shown that *RRAD* knockout can leads to an increased expression of LTCC in cardiomyocytes, which can in turn results in an increased cytoplasmic calcium level. To reveal the consequences of elevated intracellular calcium toward downstream molecules, we performed a western blot to determine which downstream pathway is affected by the change. Results as shown in Figures 6G,H, expression of activated (phosphorylated) CaMKII (p-CaMKII, CaMKII-Thr286), a strong inducer of hypertrophy pathways (Zhang et al., 2003), was significantly increased. However, expression of calcineurin, another common activator for inducing hypertrophy, did not show any alteration. Altogether, these findings implicated that Ca^2 +^-calmodulin-CaMKII may be the key pathway in mediating cardiomyocytes hypertrophy of RAD deficiency, whereas calcineurin does not play a major role.

### Management of Calcium Dysregulation Prevents the Development of Cardiac Hypertrophy

**FIGURE 7:**
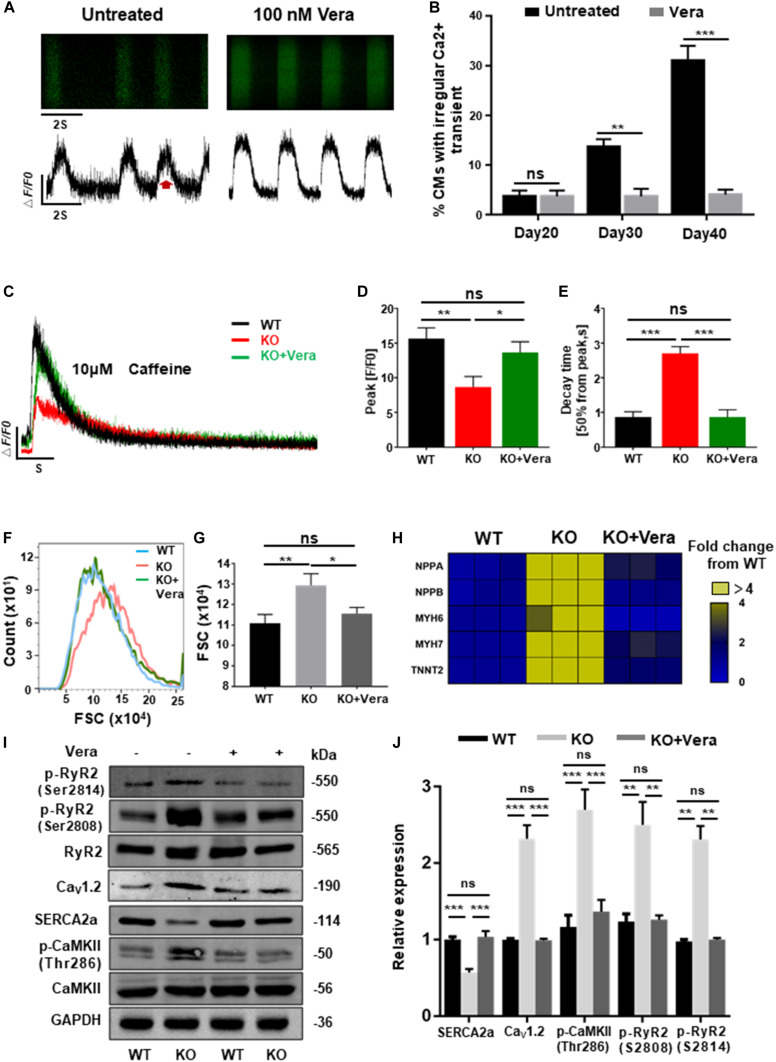
Calcium channel blocker prevents the development of cardiac hypertrophy in RAD deficient CMs. **(A)** Representative line-scan images of KO CMs treated with 100 nM verapamil for 10 days. **(B)** Quantification of percentages of KO CMs found to exhibit abnormal calcium regulation after treatment with verapamil (*n* = 30 cells per group). **(C)** Representative Ca^2 +^ transient induced by caffeine of KO CMs after treated with verapamil (*n* = 5 cells per group). **(D,E)** Peak amplitudes and decay time of KO CMs after treated with verapamil. **(F,G)** Flow cytometry shows changes in cell size of WT, KO and verapamil treated KO CMs. **(H)** Heatmap shows expression changes of genes related to hypertrophy in WT, KO, and verapamil treated KO CMs. **(I,J)** Western blot shows expression of Ca_v_1.2, SERCA2a, p-RyR2 (Ser2808, Ser2814), and p-CaMKII (Thr286) in WT, KO, and verapamil KO CMs. Quantification of protein expression normalized by GAPDH. Vera, verapamil. Data are expressed as means ± S.E.M. of three independent experiments. ns, not significant; *P < 0.05; **P < 0.01; ***P < 0.001.

Previous data have shown that elevated *I*_Ca–L_ of cardiomyocytes and dysregulation of intracellular calcium are the mechanisms responsible for cardiac hypertrophy caused by RAD deficiency. In order to block this pathological process, both WT and KO CMs at day 20 post-induction were treated with verapamil (LTCC blocker) at therapeutic dosages (100 nM). Starting after 10 days of pretreatment, homeostasis of calcium handling (Figures 7A,B), recovered storage of SR calcium (Figures 7C–E and Supplementary Figure S7A), and significantly decreased cell size (Figures 7F,G) could be observed in the treated KO CMs. Consistently, expression of several representative cardiac hypertrophy genes has shown no significant difference to WT after pharmaceutical inhibition of excessive calcium entry at early stage (Figure 7H and Supplementary Figure S7B). In addition, results of western blot shown the expression of SERCA2a, Ca_v_1.2, and p-RyR2 (RyR2-Ser2808, RyR2-Ser2814) were significantly restored. More importantly, expression of p-CaMKII (CaMKII-Thr286), a strong inducer in mediating cardiac hypertrophy, also showed a significant decrease (Figures 7I,J). Altogether, these findings suggested that early treatment of calcium dysregulation prevents the development of cardiac hypertrophy caused by RAD deficiency.

## Discussion

**FIGURE 8:**
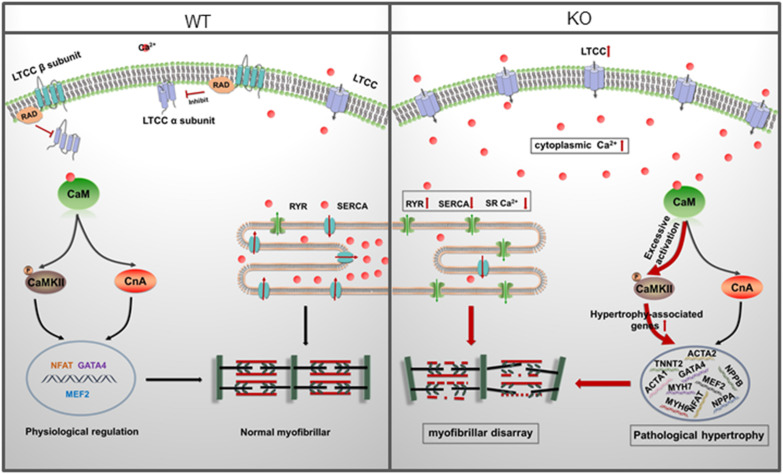
A model for mechanisms of cardiac hypertrophy in RAD deficient CMs. RAD deficiency triggers hypertrophy-associated pathway via modulation of the cytoplasmic Ca^2 +^. NCX, sodium-calcium exchanger; LTCC, L-type calcium channel; CaM, Calmodulin; CaMKII, calmodulin-dependent kinase II; CnA, Calcineurin; SR, sarcoplasmic reticulum.

In the present study, we reported an *in vitro* RAD deficient cardiomyocyte model derived from *RRAD*^–/–^ hESCs using CRISPR/Cas9 for the first time. Based on this model, functional changes, underlying mechanisms and potential therapies of RAD-deficiency could be well studied in a human cardiac background. After verification by multiple methods, we found that RAD deficient cardiomyocytes exhibited significant hypertrophic phenotypes and activated hypertrophy-associated genes. Further investigation revealed that increased LTCC expression in the membrane of cardiomyocytes and abnormal regulation of intracellular calcium level were the central mechanism of the phenotypes. Management of calcium dysregulation with LTCC blocker, verapamil, prevents the development of cardiac hypertrophy caused by RAD-deficient CMs (Figure 8).

Ras associated with diabetes, a member of the RGK subfamily in the Ras-related GTPase (Wang et al., 2010), is encoded by *RRAD* gene and abundantly expressed in cardiomyocytes (Reynet and Kahn, 1993; Maguire et al., 1994). It comprises multiple functional domains, which can interact with a variety of signal transduction molecules including Rho kinase, calmodulin, and 14-3-3 protein. In addition, RAD could impact various aspects of cardiac physiological functions and pathological processes such as cytoskeletal regulation, neointimal formation after balloon injury in fibroblastic cells, and diabetic cardiomyopathy (Ward et al., 2002; Beguin et al., 2005; Fu et al., 2005; Ilany et al., 2006). Previous studies on mice have demonstrated that deficiency of RAD significantly increases stress-induced (thoracic transverse aortic constriction or phenylephrine) cardiac hypertrophy and remodeling both *in vivo* and *in vitro* (Chang et al., 2007). However, our data demonstrated that cardiomyocytes derived from *RRAD*^–/–^ hESCs can spontaneously develop hypertrophic phenotypes such as increased size of cardiomyocytes, multi-nucleation, and disorganized arrangement of myofibrils 40 days post-induction without external factors. We speculate for two reasons. First, our cardiomyocytes are cultured at the cellular level in 2D mode, and lack the overall level of organ and system interaction. The course of cardiac hypertrophy is a slowly progressing process and is affected by various factors throughout the body. However, our model cannot simulate the pathological process at the tissue and organ level, which is also one of the limitations of this paper. Second, due to species differences, the electrophysiological characteristics of human and mice will also differ significantly, and the biological functions performed by them will also be different, which may be another reason for this result.

Calcium (Ca^2 +^) is a universal intracellular signaling molecule and is essential for the maintenance of normal functioning of the heart (Wang et al., 2010). Upon depolarization of the cardiomyocyte membrane, LTCC located on T-tubules (invaginations of the plasma membrane of the cell) is activated to allow Ca^2 +^ to enter the cytoplasm, which triggers a more substantial SR calcium release (known as Ca^2 +^-induced Ca^2 +^ release, CICR) and thus initiate a series of physiological activities such as electrophysiology, EC couppling, energy consumption (Servili et al., 2018). Consistently, on repolarization of the cardiomyocyte membrane, elevated cytoplasmic Ca^2 +^ are restored to resting state through various routes such as sarcoplasmic reticulum SERCA (SR Ca^2 +^ ATPase, transported cytoplasmic Ca^2 +^ to the SR), cell membrane sodium-calcium exchangers (NCX) and calcium pumps (NCX and Ca^2 +^ ATPases, transported cytoplasmic Ca^2 +^ to the extracellular). However, when this balance is disrupted by various factors resulting irregular Ca^2 +^ regulations or abnormally elevated intracellular Ca^2 +^ levels, it may become the initiator for many cardiac diseases such as cardiac hypertrophy or arrhythmia (Monteiro da Rocha et al., 2016; Dewenter et al., 2017). Previous studies have revealed that Rad can act as a negative regulator of LTCC activity by directly binding to their β-subunit and overexpression of S105N Rad (dominant negative mutant Rad) in guinea pig ventricular cardiomyocytes can lead to an increased intracellular Ca^2 +^ via upregulation of LTCC expression in the plasma membrane (Finlin et al., 2003; Yada et al., 2007). In our study, cardiomyocytes derived from *RRAD*^–/–^ hESCs exhibited significantly increased expression of *CACNA1C* and Ca_v1.2_ (gene and protein of LTCC α1c subunit, respectively) on transcription and protein levels, which lead to an increased *I*_Ca–L_ (pA/pF). Measurement of intracellular Ca^2 +^ regulation has shown that significant abnormal Ca^2 +^ transient such as events associated with arrhythmia-like voltage waveforms were more frequently appeared in cardiomyocytes derived from *RRAD*^–/–^ hESCs. Accordingly, we speculate that the electrophysiology of KO CMs should also be changed, but for some reason, we failed to record the action potentials of hESC-CMs. In addition, SR Ca^2 +^ stores induced by caffeine showed a smaller calcium release and prolonged decay time in KO CMs supported another finding of impaired function of calcium regulation. Moreover, severely exacerbate presentation of multiple events induced by positive inotropic PE frequently observed in KO CMs were also compatible with the clinical manifestations of patients with cardiac hypertrophy. Interestingly, all these abnormities above occur prior to the onset of hypertrophic phenotype indicated that overexpressed LTCC and abnormal Ca^2 +^ handling may be the initiator of cardiac hypertrophy caused by RAD deficiency.

As an important intracellular second messenger, activity-induced Ca^2 +^ influx through LTCC can initiate multiple Ca^2 +^-dependent signaling cascades and then lead to the activation of specific transcription programs, a process known as excitation-transcription (ET) coupling which play a critical role not only in cardiac homeostasis but also in cardiac disease development, since diseased cardiomyocytes show vast alterations in Ca^2 +^-handling and Ca^2 +^-dependent transcriptional patterns (Kim and Kim, 2018). Among these, calmodulin, CaMKII, and calcineurin are well-studied signaling molecules in cardiomyocytes (Dewenter et al., 2017). Generally, Ca^2 +^ entering the cardiomyocytes initially binds to calmodulin and then activates Ca^2 +^-dependent cascades in the form of Ca^2 +^-calmodulin complex, such as Ca^2 +^-calmodulin-CaMKII and Ca^2 +^-calmodulin-calcineurin pathways, which are major signal mediators of cardiac hypertrophy and remodeling (Lehman et al., 2018). In addition, Ca^2 +^ combined with calmodulin can induce the expression of ANF via interacting with CAMTA2, known as calmodulin-binding transcriptional activator 2 (Long et al., 2014). Meanwhile, CAMTA2 can also bind to NKX2-5 and participate in the pathogenesis of cardiac hypertrophy (Dewenter et al., 2017). In our study, further investigation for time-based gene expression profiling revealed that cardiomyocytes derived from *RRAD*^–/–^ hESCs exhibited significant upregulation of hypertrophy-associated genes which occurred right after the disorder of intracellular Ca^2 +^ homeostasis. Also, increased cell death in the *RRAD*^–/–^ cardiomyocytes may be a result of increased intracellular Ca^2 +^ (Harvey and Leinwand, 2011; Dewenter et al., 2017). Furthermore, the increased expression of p-CaMKII, a potent inducer of hypertrophy pathways, revealed a critical role for the Ca^2 +^-calmodulin-CaMKII pathway in the hypertrophic phenotype caused by RAD deficiency.

A majority of patients with cardiac hypertrophy have varying degrees of cardiac dysfunction, usually caused by the thickening ventricular wall, which could lead to notably elevated ventricular pressures (Maron et al., 2003, 2014). ANF (atrial natriuretic factor) and BNP (brain natriuretic peptide), are diagnostic indicators of cardiac function secreted by atrial and ventricular myocytes, respectively. Both ANF and BNP are elevated more than 100 times in patients with cardiac hypertrophy to compensate for increased blood volume and pressure caused by thickening of the ventricular wall (Rodeheffer et al., 1986; Gardner, 2003; Taylor et al., 2018). Consistently, our data has shown that the expression levels of *NPPA* and *NPPB* (coding genes for ANF and BNP, respectively) are significantly increased, which reflects the impaired function of cardiomyocytes caused by RAD deficiency to some extent. In addition, upregulated β-myosin expression (β-MHC, encoded by *MYH7*), accompany with downregulated α-myosin expression (α-MHC, encoded by *MYH6*) is a sensitive indicator of cardiac hypertrophy (Lowes et al., 1997; Miyata et al., 2000). Furthermore, the shift toward β-MHC may also be the main cause of decreased contractile function and prolonged relaxation of cardiomyocytes during the transition from compensatory cardiac hypertrophy to end stage of heart failure (Nagata et al., 1998). Our data has shown that expression of *MYH7* is significantly increased in cardiomyocytes derived from *RRAD*^–/–^ hESCs, both at the transcriptional and protein level. The result is also consistent with the pathological process in patients with cardiac hypertrophy.

The relationship between disorders of intracellular Ca^2 +^ and cardiac hypertrophy has been confirmed for over a decade (Fatkin et al., 2000). Also, our data demonstrated that abnormal Ca^2 +^ handling caused by overexpressed LTCC in cardiomyocytes may be the initiator of cardiac hypertrophy caused by RAD deficiency. Subsequently, inhibiting calcium dysregulation at early stage could prevent the development of cardiac hypertrophy further reinforce the hypothesis above.

However, it is important to notice that there are some limitations in our study. Firstly, maturity is critical for studying the function of hESC-CMs. Genetically modified hPSCs have been commonly used to study the gene function in cardiomyocytes, indicating that these cells are excellent models for cardiovascular research (Lan et al., 2013; Fogarty et al., 2017; Mosqueira et al., 2018; Li et al., 2019). In this study, we found that *RRAD*^–/–^ cardiomyocytes were more prone to hypertrophic phenotype, whereas, WT cardiomyocytes were also exhibited a certain phenotype such as low frequencies of myofibrillar disarray, multi-nucleation under the same culture conditions. This may be closely related to the immaturity of hESC-CMs. Therefore, more technologies aimed at promoting the maturation of hESC-CMs, such as increasing culture time (Kamakura et al., 2013), mechanical stimulation (Mihic et al., 2014), and tissue engineered tissues (Feric and Radisic, 2016) were necessary for future research. Secondly, hESCs differentiated cardiomyocytes cannot mimic disease phenotypes at the tissue and organ levels is another limitation of this article. Even though we hypothesized that the knockout of *RRAD* would cause an abnormal increase in Ca^2 +^ in the cytoplasm of cardiomyocytes, activate the hypertrophy-associated Ca-CaM-CaMKII pathway, and result in increased expression of a series of hypertrophy genes including *NPPA*, *NPPB*, future researches aimed at the overall level will be necessary (Carvajal-Vergara et al., 2010; Burridge et al., 2012). Despite this, our study reported potential functional changes, underlying mechanisms associated with RAD-deficiency for the first time in a human cardiac background which may provide novel insights for the study of the pathogenesis and treatment of cardiac hypertrophy.

## Data Availability Statement

The raw data generated during this study can be found in the SRA database, accession number PRJNA661724.

## Author Contributions

FL, YW, XT, YL, and XlL designed the experiments. YL, XwL, FW, RB, YZ, SM, TD, YC, SZ, and W-JL performed the experiments. YL, XlL, XwL, FW, XT, YZ, YC, and W-JL analyzed the data. YL, FL, JG, XlL, YZ, and YC wrote the manuscript. All authors contributed to the article and approved the submitted version.

## Conflict of Interest

The authors declare that the research was conducted in the absence of any commercial or financial relationships that could be construed as a potential conflict of interest.

## Footnotes

1 http://crispr.mit.edu
