# Supplementary material for: RAD-Deficient Human Cardiomyocytes Develop Hypertrophic Cardiomyopathy Phenotypes Due to Calcium Dysregulation
Source: Front Cell Dev Biol. 2020 Oct 22;8:585879. doi: 10.3389/fcell.2020.585879 (PMC7642210; doi:10.3389/fcell.2020.585879)
Supplement: Supplementary file 1 [file Data_Sheet_1.pdf]

Table S1. Primer sequences used for q-PCR

Gene	Forward 5'-3'	Reverse 5'-3'
NPPA	ACAATGCCGTGTCCAACGCAGA	CTTCATTCGGCTCACTGAGCAC
NPPB	TCTGGCTGCTTTGGGAGGAAGA	CCTTGTGGAATCAGAAGCAGGTG
MYH6	TCTCCGACAACGCCTATCAGTAC	GTCACCTATGGCTGCAATGCT
MYH7	GGCAAGACAGTGACCGTGAAG	CGTAGCGATCCTTGAGGTTGTA
ACTA1	AGGTCATCACCATCGGCAACGA	GCTGTTGTAGGTGGTCTCGTGA
ACTA2	CTATGCCTCTGGACGCACAACT	CAGATCCAGACGCATGATGGCA
MYL2	TACGTTCGGGAAATGCTGAC	TTCTCCGTGGGTGATGATG
MYL7	CCGTCTTCCTCACGCTCTT	TGAACTCATCCTTGTTCACCAC
TNNT2	TTCACCAAAGATCTGCTCCTCGCT	TTATTACTGGTGTGGAGTGGGTGTGG
CX43	GGAGATGAGCAGTCTGCCTTTC	TGAGCCAGGTACAAGAGTGTGG
SCN5A	CTGACCTCACCATCACTATGTG	GCTGTGAAAATCCCTGTGAAG
KCNA5	GTTCCGCATCTTCAAGCTCTCC	CGAAGTAGACGGCACTGGAGAA
KCNH2	AATCGCCTTCTACCGGAAAG	CACCATGTCCTTCTCCATCAC
KCNQ1	TCTGTCTTTGCCATCTCCTTC	CCTCCATGCGGTCTGAATG
KCNJ2	AAGACGGTATGAAGTTGGCC	CGGGTGTGGACTTTACTCTTC
CACNA1C	CAGAGGCTACGATTTGAGGA	GCTTCACAAAGAGGTCGTGT
ATP2A2	GATCACACCGCTGAATCTG	AGTATTGCGGGTTGTTCCAG
PLN	AGCACGTCAAAAGCTACAGAATCT	CTGATGTGGCAAGCTGCAGATC
RYR2	AGAACTTACACACGCGACCTG	CATCTCTAACCGGACCATACTGC
ATP2B4	CTCACCGAACTGACCTGTATCG	GGCTGTGTTGATGTTGTCACCTG
NCX1	TCGAGATTGTCTCTTCGGGC	CATATGCAAACACCGAGGCG
COL1A1	GATTCCCTGGACCTAAAGGTGC	AGCCTCTCCATCTTTGCCAGCA
COL4A1	TGTTGACGGCTTACCTGGAGAC	GGTAGACCAACTCCAGGCTCTC
CASP3	GGTATTGAGACAGACAGTGG	CATGGGATCTGTTTCTTTGC
BCL2	ATCGCCCTGTGGATGACTGAGT	GCCAGGAGAAATCAAACAGAGGC
BAX	TCAGGATGCGTCCACCAAGAAG	TGTGTCCACGGCGGCAATCATC
CALM1	CCAACAGAAGCTGAATTGCAGGA	CAAAGACTCGGAATGCCTCACG
PPP3CA	GCCCTGATGAACCAACAGTTCC	GCAGGTGGTTCTTTGAATCGGTC
CAMK2D	ACACGGTGACTCCTGAAGCCAA	GTCTCCTGTCTGTGCATCATGG
MEF2A	CAAGGGCATGATGCCTCCACTA	GCTGAGTACACAAGTCCTTGCG
GATA4	GCGGTGCTTCCAGCAACTCCA	GACATCGCACTGACTGAGAACG
NFATC4	GCACCGTATCACAGGCAAGATG	TCAGGATTCCCGCGCAGTCAAT
CACNB2	TTCCATGCGACCAGTGGTCCTA	GCGTTTGGCAAGCGAGATGTCA
ITPR1	GTGACAGGAAACATGCAGACTCG	CAGCAGTTGCACAAAGACAGGC
GAPDH	GGAGCGAGATCCCTCCAAAAT	GGCTGTTGTCATACTTCTCATGG
