# Supplementary material for: RAD-Deficient Human Cardiomyocytes Develop Hypertrophic Cardiomyopathy Phenotypes Due to Calcium Dysregulation
Source: Front Cell Dev Biol. 2020 Oct 22;8:585879. doi: 10.3389/fcell.2020.585879 (PMC7642210; doi:10.3389/fcell.2020.585879)
Supplement: Supplementary file 2 [file Data_Sheet_2.pdf]

Table S2. Primary and Secondary Antibodies

Type	Antibody	Application	Dilution	Species	Manufacturer And Catalog Number
Primary	Anti-OCT4	Immunofluorescence	1:100	Rabbit Polyclonal	Santa Cruz sc-9081
	Anti-SSEA4	Immunofluorescence	1:100	Mouse Monoclonal	Santa Cruz sc-21704
	Anti-MLC2a	Immunofluorescence	1:100	Mouse Monoclonal	Santa Cruz sc-365255
	Anti-MLC2v	Immunofluorescence	1:50	Rabbit Polyclonal	Proteintech 10906-1-AP
	Anti-Rad GTPase	Western blot	1:1000	Mouse Monoclonal	Santa Cruz sc-373988
	Anti-α-actinin	Immunofluorescence	1:100	Rabbit Polyclonal	Abcam Ab137346
		Western blot	1:1000		
	Anti-cTnT	Immunofluorescence	1:100	Mouse Monoclonal	Abcam Ab8295
		Western blot	1:1000		
		Flow cytometry	1:200		
	Anti-MYH7	Western blot	1:1000	Mouse Monoclonal	Abcam Ab 174640
	Anti-Bcl2	Western blot	1:1000	Rabbit Polyclonal	Proteintech 12789-1-AP
	Anti-Bax	Western blot	1:1000	Rabbit Polyclonal	Proteintech 50599-2-AP
	Anti-SERCA2a	Western blot	1:1000	Rabbit Monoclonal	Cell signaling #9580
	Anti-PLN	Western blot	1:1000	Rabbit Polyclonal	Cell signaling #8495
	Anti-L-type Ca^{2+} CP α1C	Western blot	1:1000	Mouse Monoclonal	Santa Cruz sc-398433
	Anti-L-type Ca^{2+} CP β2	Western blot	1:1000	Mouse Monoclonal	Santa Cruz sc-81890
	Anti-Phospho-CaMKII(Thr286)	Western blot	1:1000	Rabbit Monoclonal	Cell signaling #12716
	Anti-CaMKII	Western blot	1:1000	Rabbit Monoclonal	Abcam Ab52476
	Anti- Calcineurin	Western blot	1:1000	Rabbit Polyclonal	Cell signaling #2614
	Anti-GAPDH	Western blot	1:1000	Mouse Monoclonal	Santa Cruz sc-365062
	Anti-IP3R-1	Western blot	1:1000	Mouse Monoclonal	Santa Cruz sc-271197
	Anti-RyR2	Western blot	1:1000	Mouse Monoclonal	Santa Cruz sc-376507
	Anti-Phospho-RyR2(Ser2808)	Western blot	1:1000	Rabbit Polyclonal	Abcam Ab59225
	Anti-Phospho-RyR2(Ser2814)	Western blot	1:1000	Rabbit Polyclonal	Invitrogen #PA5-104558
	Anti-alpha 1 Sodium Potassium	Western blot	1:1000	Mouse Monoclonal	Abcam Ab7671
Secondary	Goat anti-Mouse IgG Alexa Fluor 594	Immunofluorescence	1:200	Goat anti-Mouse IgG	Invitrogen A21145
	Goat anti-Rabbit IgG Alexa Fluor 488	Immunofluorescence	1:200	Goat anti-Rabbit IgG	Invitrogen A32731
	Chicken anti-Rabbit IgG Alexa Fluor 594	Immunofluorescence	1:200	Chicken anti-Rabbit IgG	Invitrogen A21442
	Chicken anti-Mouse IgG Alexa Fluor 488	Immunofluorescence	1:200	Chicken anti-Mouse IgG	Invitrogen A21200

	Goat anti-Rabbit IgG (H + L) IRDye 800CW	Western blot	1:20000	Goat anti-Rabbit IgG	LI-COR 926-32211
	Goat anti-Mouse IgG (H + L) IRDye 800CW	Western blot	1:20000	Goat anti-Mouse IgG	LI-COR 926-32210
