# Supplementary figures and images for: RAD-Deficient Human Cardiomyocytes Develop Hypertrophic Cardiomyopathy Phenotypes Due to Calcium Dysregulation
Source: Front Cell Dev Biol. 2020 Oct 22;8:585879. doi: 10.3389/fcell.2020.585879 (PMC7642210; doi:10.3389/fcell.2020.585879)

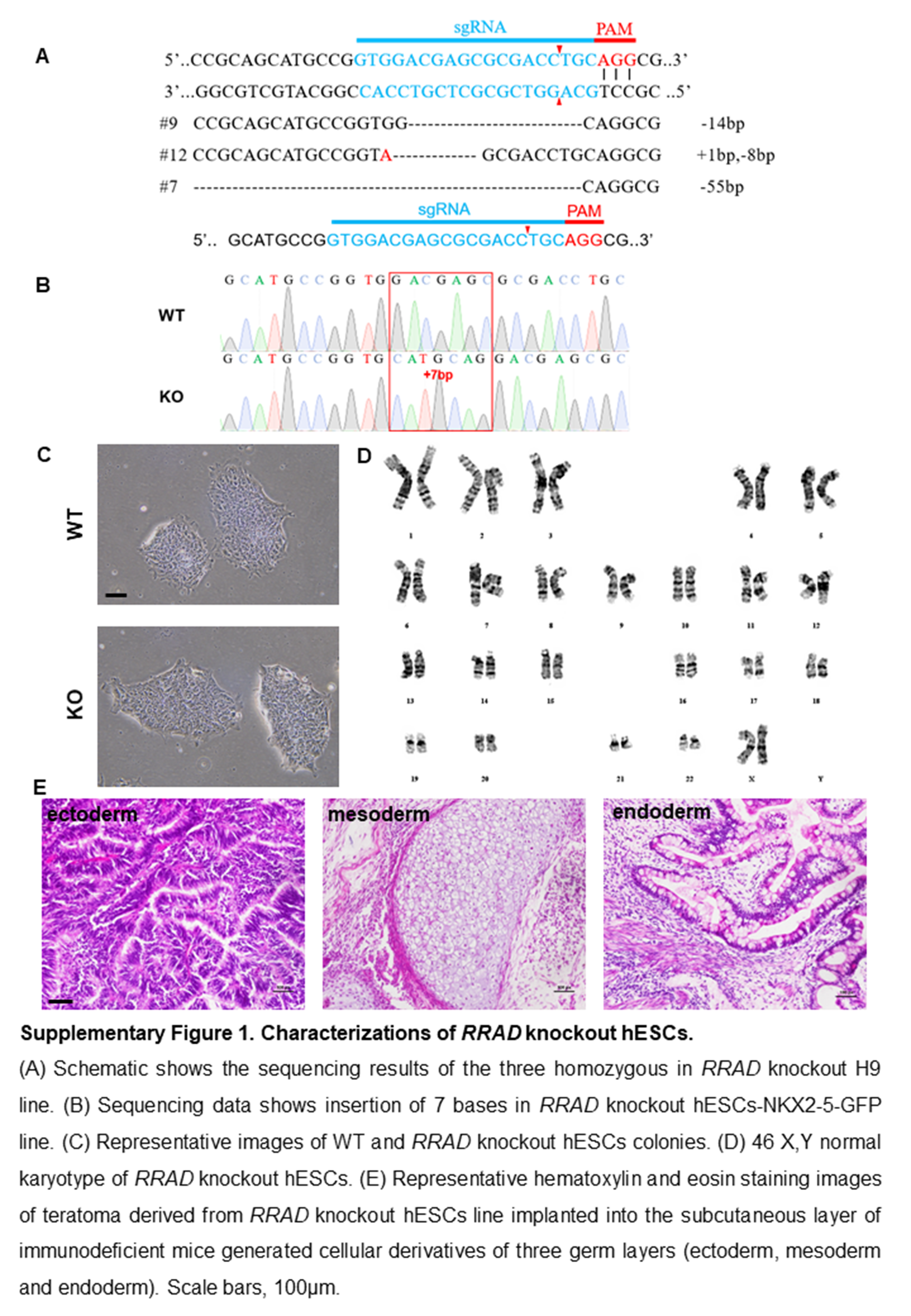

Supplement: Supplementary file 3 [file Image_1.tif]

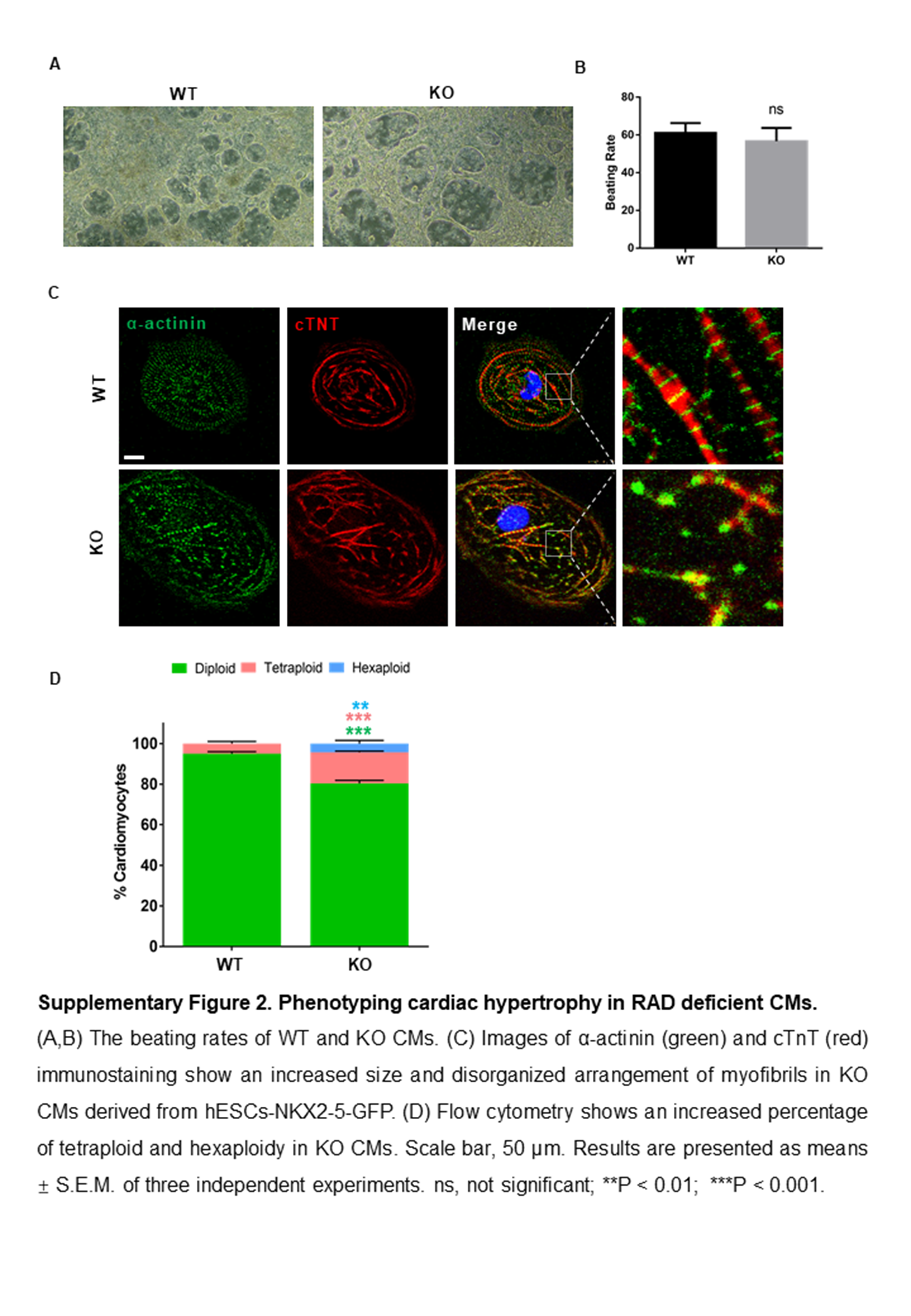

Supplement: Supplementary file 4 [file Image_2.tif]

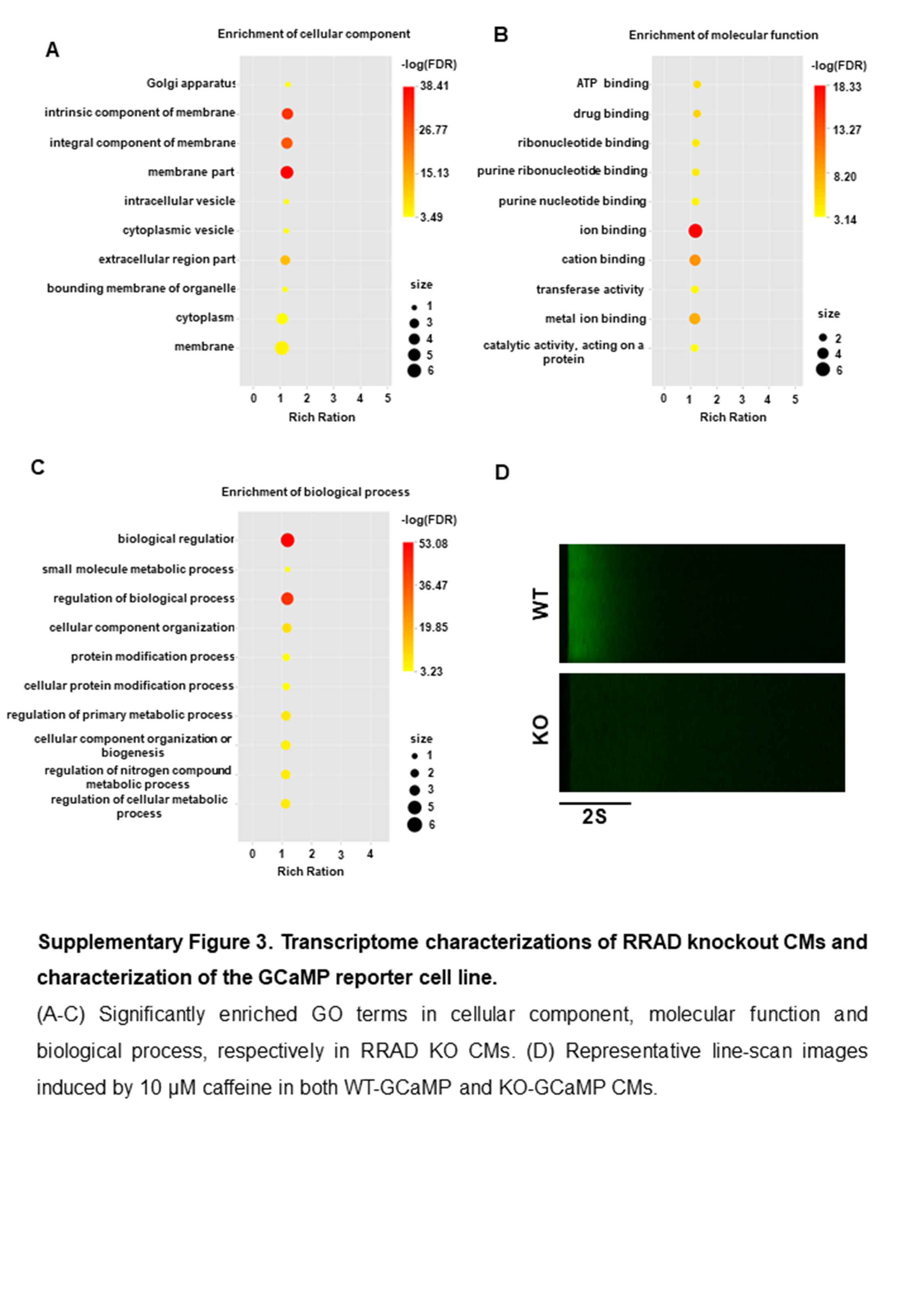

Supplement: Supplementary file 5 [file Image_3.tif]

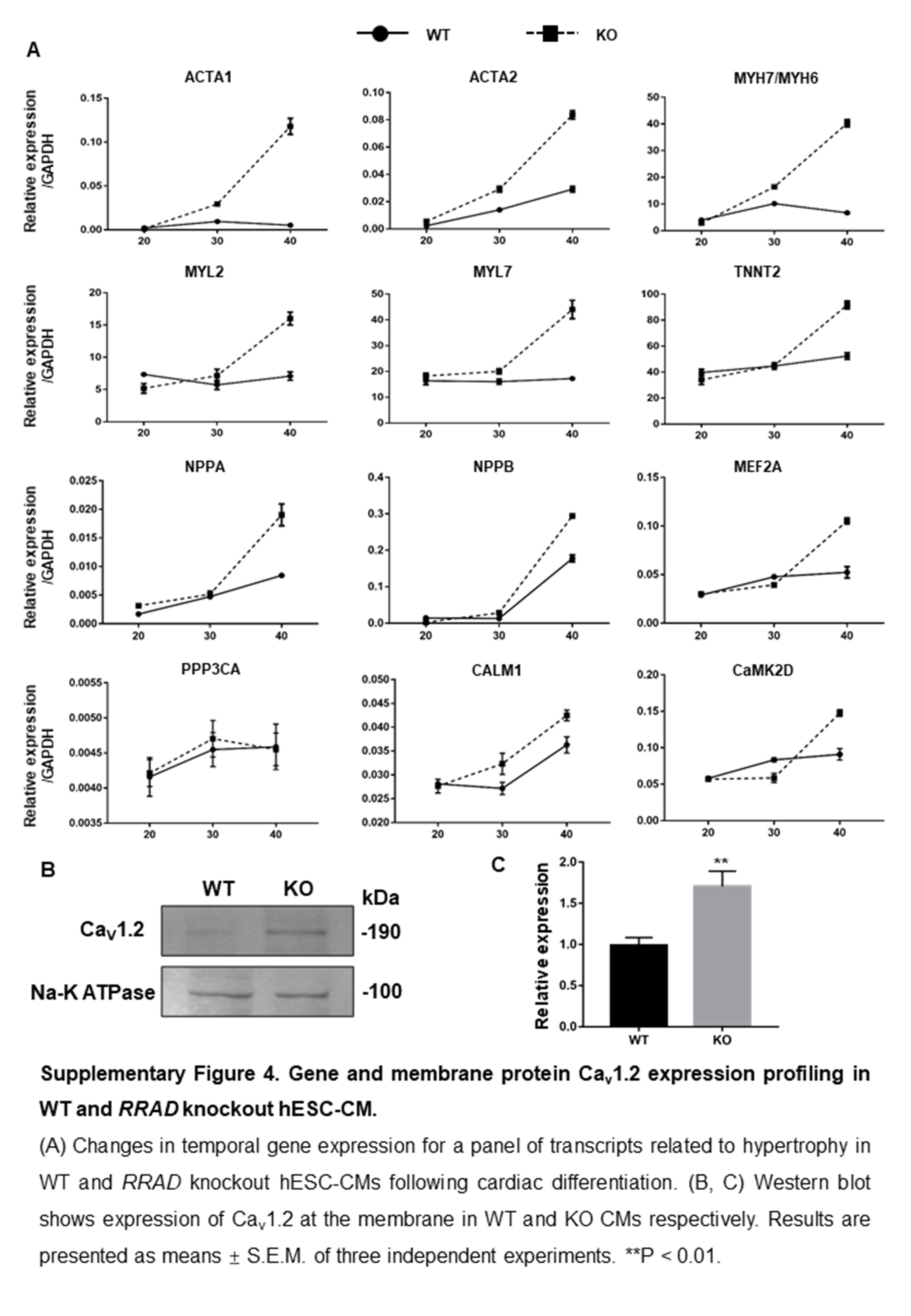

Supplement: Supplementary file 6 [file Image_4.tif]

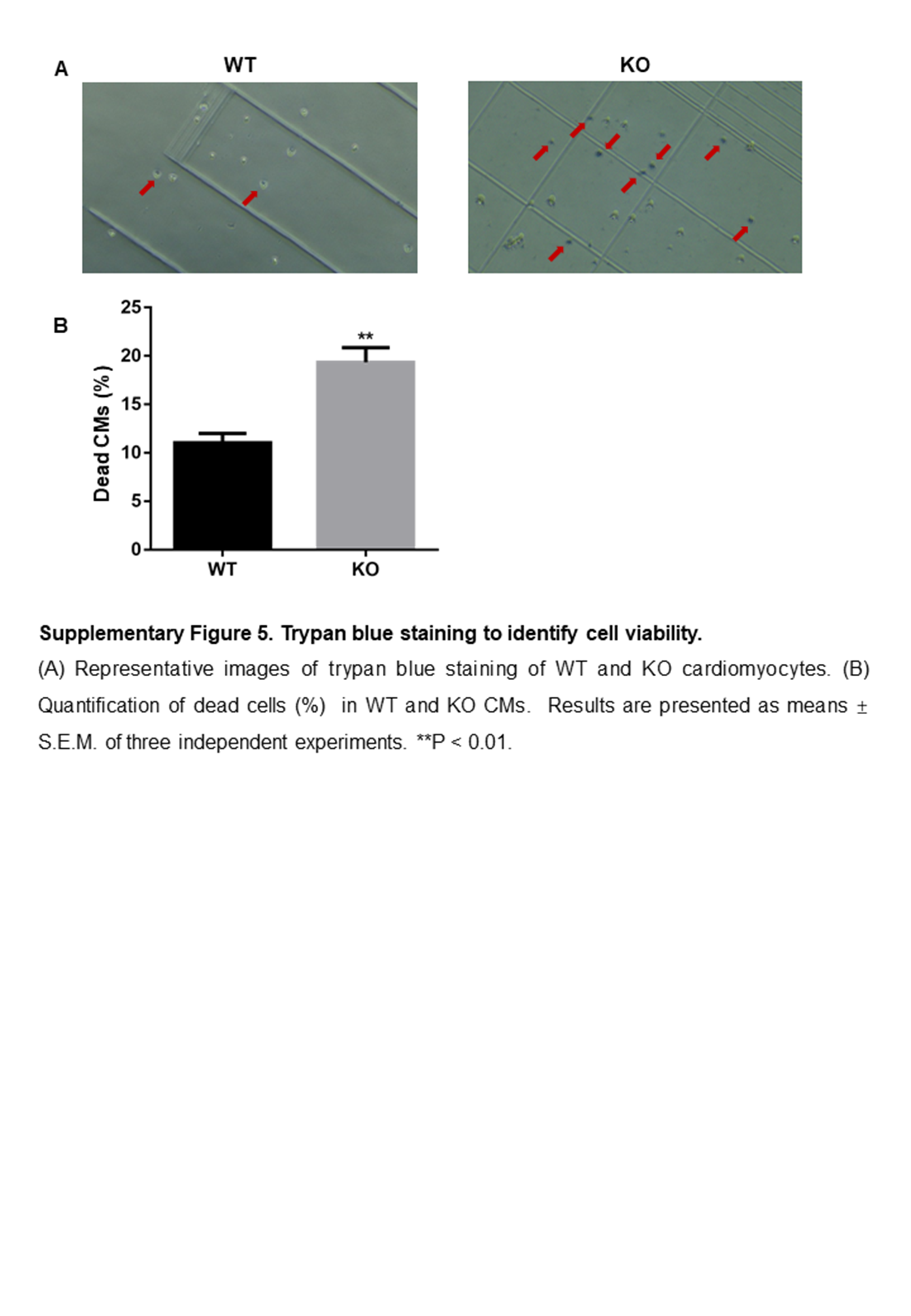

Supplement: Supplementary file 7 [file Image_5.tif]

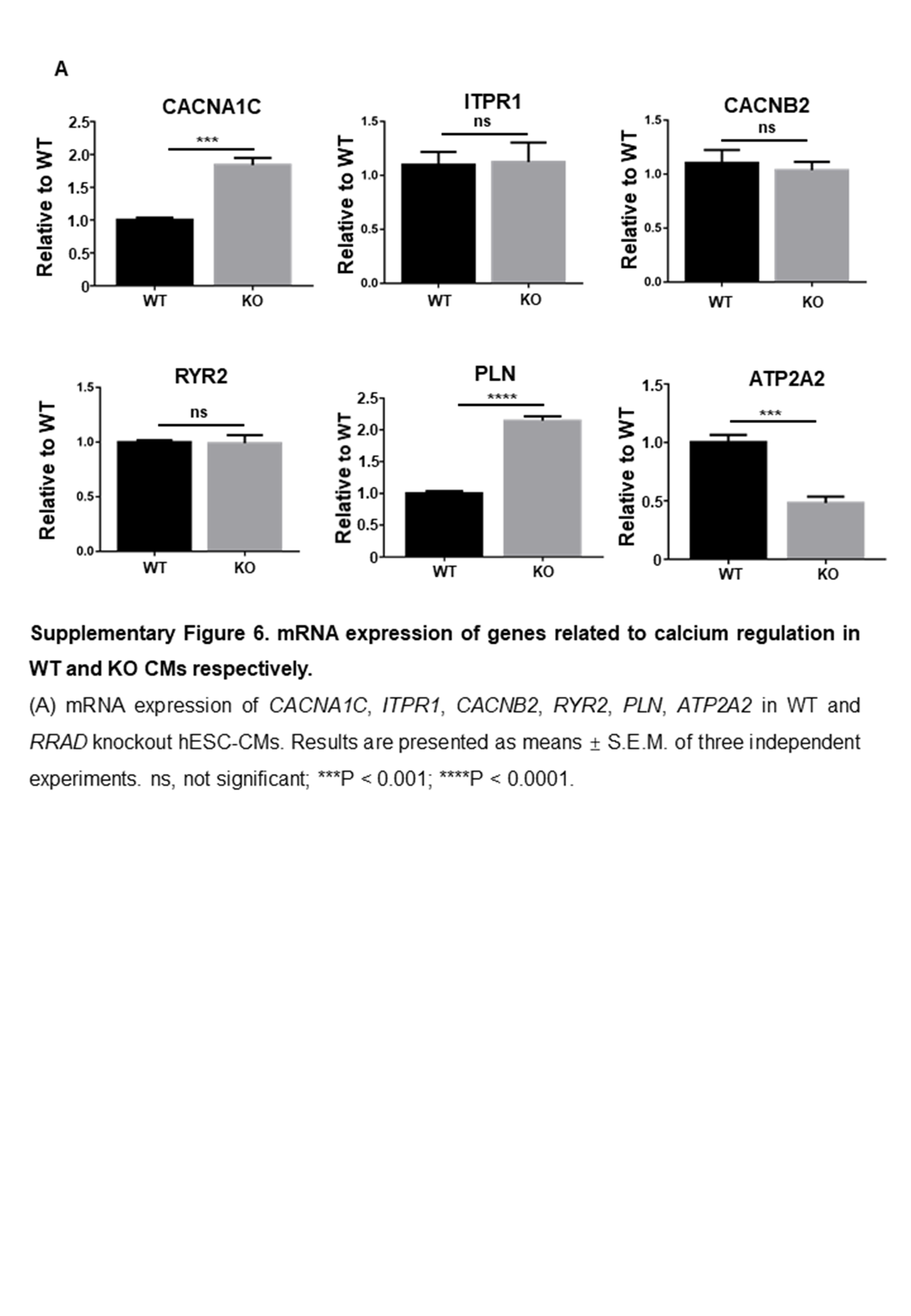

Supplement: Supplementary file 8 [file Image_6.tif]

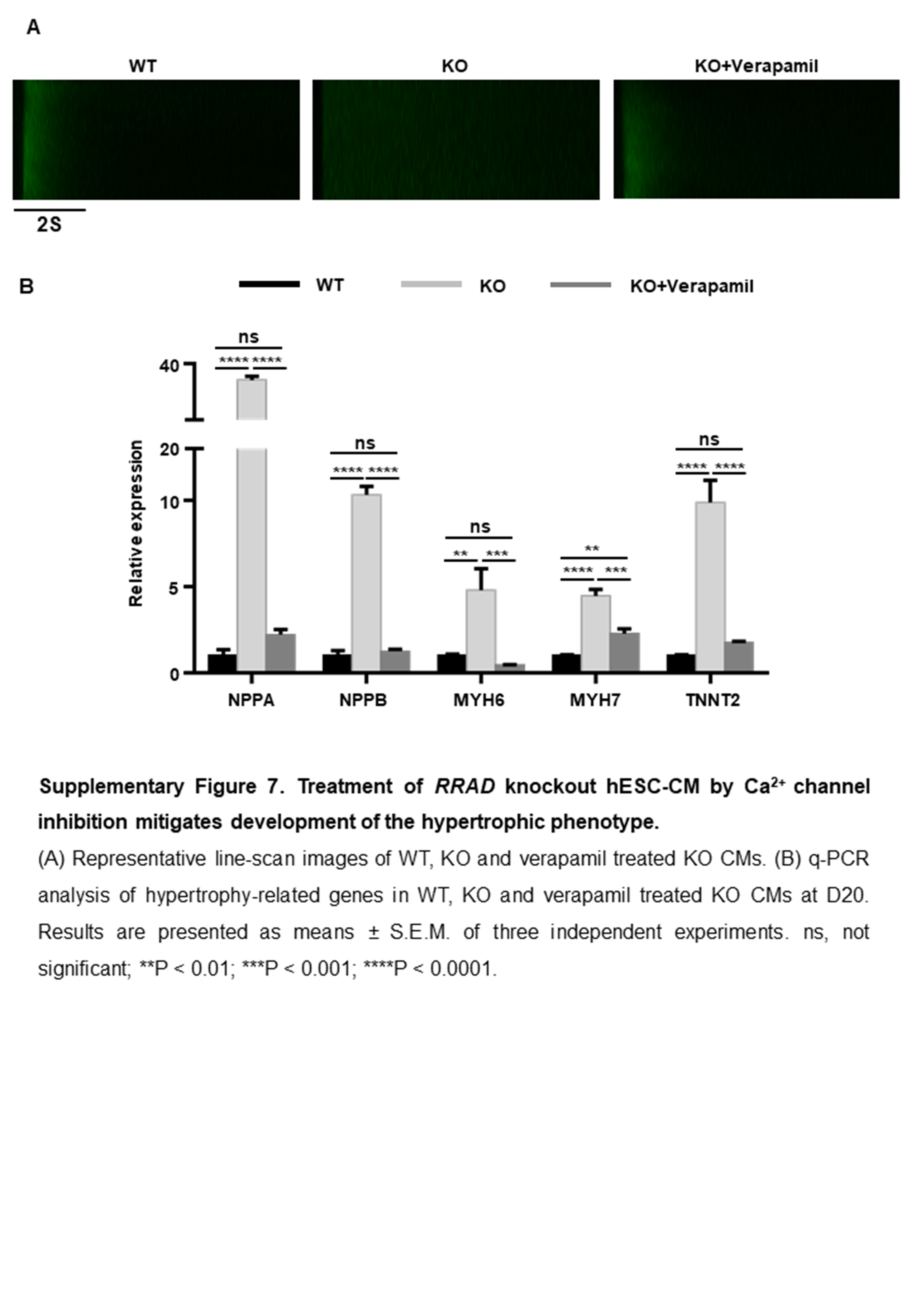

Supplement: Supplementary file 9 [file Image_7.tif]
